# An Infrastructure for Enabling Dynamic Fault Tolerance in Highly-Reliable Adaptive Distributed Embedded Systems Based on Switched Ethernet

**DOI:** 10.3390/s22187099

**Published:** 2022-09-19

**Authors:** Alberto Ballesteros, Manuel Barranco, Julián Proenza, Luís Almeida, Francisco Pozo, Pere Palmer-Rodríguez

**Affiliations:** 1 Departament de Matemàtiques i Informàtica, Universitat Illes Balears, 07122 Palma de Mallorca, Spain; 2 CISTER Research Center in Real-Time and Embedded Computing Systems, 4200-135 Porto, Portugal; 3 FEUP Faculdade de Engenharia da Universidade do Porto, 4200-465 Porto, Portugal; 4 Hitachi Energy, 72226 Vasteras, Sweden

**Keywords:** DFT4FTT, distributed, embedded, adaptivity, dependability, reliability, fault tolerance, dynamic fault tolerance, resilience

## Abstract

Distributed Embedded Systems (DESs) carrying out critical tasks must be highly reliable and hard in real-time. Moreover, to operate in dynamic operational contexts in an effective and efficient manner, they must also be adaptive. Adaptivity is particularly interesting from a dependability perspective, as it can be used to develop dynamic fault tolerance mechanisms, which, in combination with static ones, make it possible to provide better and more efficient fault tolerance. However, constructing a DES with such complexity presents many challenges. This is because all the mechanisms that support fault tolerance, real-time, and adaptivity must be designed to operate in a coordinated manner. This paper presents the Dynamic Fault Tolerance for Flexible Time-Triggered Ethernet (DFT4FTT), a self-reconfigurable infrastructure for implementing highly reliable adaptive DES. Here, we describe the design of its hardware and software architecture and the main set of mechanisms, with a focus on fault tolerance.

## 1. Introduction

Distributed Embedded Systems (DESs) play a key role and are almost ubiquitous in many economic sectors, such as civil avionics, the automotive industry, railway signaling, road transportation, healthcare, energy distribution, and telecommunications. This type of system is a combination of hardware and software. In a DES, the hardware is constituted by computational elements called *nodes*, which are interconnected through a network that includes multiple *links*. As for the software, it is typically implemented as a set of functional elements called *tasks* that are executed on the nodes, that coordinate by exchanging *messages*, and that cooperate to achieve some common goal.

DESs are mostly used to interact with the real world, where the specific instant in which an action is carried out has a huge impact on its outcome. That is why DESs typically have real-time requirements. A system is said to have real-time restrictions if its correct operation depends on its ability to provide a correct response before some *deadline*. Moreover, in some environments, a DES failing to provide a correct service may have catastrophic consequences. DESs operating in such environments must guarantee a trustworthy service, that is, they must be dependable. Dependability is a broad concept that contains several attributes. Among them, in this work, we address the *reliability*, that is, the ability of the system to provide a correct and continuous service [1].

**Figure 1:**
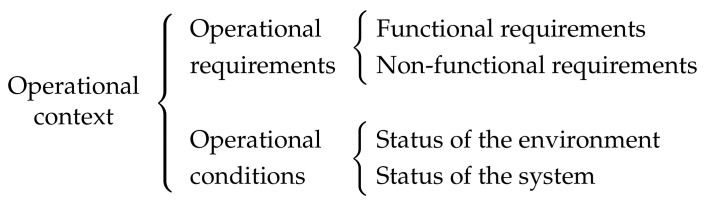
Definition of operational context.

Apart from the aforementioned requirements, nowadays there is a huge interest in DESs that are capable of operating in dynamic and unpredictable *operational contexts*. By operational context, we mean all the relevant aspects related to the operation of the system that are susceptible to change. As shown in Figure 1, this includes both the *operational requirements* and the *operational conditions*. The former can be defined as the set of functionalities the system has to carry out, the *functional requirements*, together with the real-time and reliability guarantees these functionalities have to provide, the *non-functional requirements*. The latter are the circumstances under which the system has to fulfill its operational requirements. While the *status of the environment* comprises external aspects that could affect the operation of the system, such as electromagnetic radiation, the *status of the system* captures the situation of the hardware, which can change due to permanent faults.

Traditional DESs have been designed to operate in predictable operational contexts. This has led to static approaches that guarantee that the operational requirements are met, as long as the operational context has been foreseen correctly. However, if the DES has to operate under dynamic operational contexts, these static approaches lead to an *inefficient* use of the resources. This is because static DESs are dimensioned to cope with the worst-case scenario in which they are assumed to operate. However, these scenarios are unlikely to happen, and, thus, static DESs are typically over-dimensioned. One way to overcome this limitation is through *mode change* [2]. That is, at design time the set of operational contexts are identified and, for each one of them, a *mode* of operation is defined. At runtime, a mode change protocol is responsible for applying the appropriate mode upon a change in the operational context. Consequently, at each instant, only the required tasks are loaded into the nodes, which is more efficient than a static approach. Note, however, that solutions based on static or mode-change approaches require prior knowledge of the operational contexts under which the DES is operating. That is why, if the operational context changes in an unpredictable manner, both can be *ineffective*.

To operate under dynamic and unpredictable operational contexts efficiently and effectively, the DES must be *adaptive*. An adaptive DES (ADES) has the ability to autonomously manage, with a high level of granularity, the assignment of its computational and communication resources to fulfill the operational requirements when the operational context changes. An ADES must be able to determine when its current *configuration* does not meet the operational requirements and, in response, find and apply a new configuration that does so. A system configuration can be defined as an allocation of tasks and messages to nodes and links, respectively, together with their execution and transmission attributes. Note that this approach provides a level of granularity in the management of the resources significantly higher than mode change as specific elements, like a single task or a task attribute, can be changed. Some examples of potential applications of ADESs are autonomous vehicles, exploration vehicles, smart machinery, and self-repairable devices.

Adaptivity is especially appealing from a dependability perspective as the rearrangement capacity can be used to make fault-tolerance mechanisms dynamic [3].

**Dynamic Fault Tolerance** (DFT) is the capacity of the system to dynamically manage its resources to meet the dependability requirements efficiently and effectively, despite predictable or unpredictable changes in the said requirements or the operational conditions.

It should be noted that, although the term Adaptive Fault Tolerance has been historically used to define this concept, we prefer to use the term Dynamic Fault Tolerance. This is because, following the guidelines of Årzén in [4], we consider the word adaptive to be more general and intended to be used to define the system as a whole. That is, we reserve the term adaptive to talk about the system’s ability to “modify its behavior and/or architecture to changing conditions and requirements”. Note, in this regard, that adaptivity includes the application-level intelligence that decides on the changes to carry out. In contrast, we reserve the terms flexible and dynamic to define the mechanisms that operate in the subsystems and that give support to achieve said adaptivity.

The implementation of static fault-tolerance mechanisms in combination with dynamic ones provides several benefits:

1. **Efficient use of the resources**. As already discussed, systems that use static approaches must be dimensioned to cope with the worst-case scenario in which they are assumed to operate. However, these types of scenarios are unlikely to occur and, thus, the system is built with an amount of resources far beyond the ones necessary most of the time. This is particularly noticeable in critical systems where resource redundancy is typically used to tolerate faults. By means of DFT, the system can dynamically reserve at run-time the resources that are strictly necessary for the current operational context. For example, if task replication is used to tolerate faults affecting the nodes, instead of selecting a static number of task replicas to address the worst-case scenario, this value can be dynamically selected to fulfill the reliability requirements in each specific scenario, for instance, using fewer replicas when the environment is more benign or when the reliability requirements are relaxed.
2. **Resilience**. This term [5] can be defined as “the persistence of dependability when facing changes”. These changes can be classified according to their nature, prospect, and timing. DFT can be used to make a system resilient when facing unforeseen functional and environmental changes occurring at any moment during the operation of the system. On the one hand, the number of external factors that can affect the operation of a system can be enormous or even unknown. Consequently, designing a system to cope with all their combinations using a solution based on a static or mode-change approach can be unfeasible. A system including DFT can dynamically manage fault tolerance resources with enough granularity and adjust its fault tolerance mechanisms to face operational conditions beyond those that could be faced through static or mode-change approaches. Furthermore, the higher the level of granularity in the management of these resources, the more appropriately the fault tolerance mechanisms could be adjusted. On the other hand, as will be discussed later, in dynamic operational contexts, the operational requirements can change in an unpredictable manner. This includes the non-functional requirements and, in particular, the reliability requirements. A system being able to adjust its fault tolerance resources to face these changes dynamically can not only make efficient use of these resources but also provide more effective fault tolerance. For example, if task replication is used to tolerate faults that affect nodes, the number of task replicas can be increased to meet higher reliability requirements.
3. **Survivability**. When the system must operate under extreme operational conditions, the available resources may not be sufficient to meet operational requirements. This can happen because, for instance, the accumulation of permanent hardware faults that could significantly reduce the amount of resources available and/or severe environmental conditions that could require an amount of fault tolerance resources beyond the ones available. In these scenarios, the system can move into a degraded mode [6] in which the system tries to survive as much as possible by operating with the essential services at the expense of some less critical services and/or meeting some of the reliability requirements. Although this can be implemented by means of a mode change approach by specifying predefined operation modes, DFT makes it possible for the system to find the most appropriate configuration, that is, one that maximizes the service delivery considering the available resources. This is interesting in systems such as autonomous spatial exploration probes.

Constructing an ADES with DFT features presents two main challenges. The first challenge is that it must provide a means to tune the operation of its underlying subsystems dynamically, that is, they must be *flexible*. In particular, the mechanisms that support real-time and fault-tolerance features must be flexible. For instance, in the event of a node failing, with DFT it is possible to restore the service if the tasks being executed in the said node are reallocated to other nodes. For this to happen, the system has to be able to provide enough flexibility to (1) disable the faulty node so that it cannot generate erroneous messages, (2) find a new allocation of tasks that meets the real-time requirements, and (3) start the execution of the tasks in the corresponding nodes. Moreover, if active task replication is used, replicas of the same group need to be synchronized. All of this calls for extended mechanisms like holistic online scheduling of tasks and messages, triggering the execution of tasks and transmission of messages, or reaching an agreement among task replicas when one of them is recovered.

The second challenge of constructing an ADES with DFT capabilities is that its underlying subsystems cannot be designed orthogonally, that is, considering each subsystem independently of the other ones, if we seek efficiency and effectiveness. An example of inefficiency can occur when two fault tolerance mechanisms use the same type of resources. For example, if task replication is used to tolerate software faults and link replication is used to tolerate network faults, there will be message replicas generated by the task replicas and by the link replicas. In a noncoordinated approach, the number of replicas used in one of these two mechanisms is determined without considering the other one, which leads to an inefficient use of the network resources. Regarding effectiveness, in a system with such internal complexity, mechanisms can interfere among them in a way that impairs their ability to operate correctly. For instance, two independent fault tolerance mechanisms that unintendedly react to the same event will start performing changes in the system in parallel with no coordination, potentially leaving the system in an unwanted state. Consequently, ADES must be designed following a *holistic* approach so that specific subsystems at different levels of the architecture can operate in a coordinated manner.

In this paper, we present the Dynamic Fault Tolerance for Flexible Time-Triggered Ethernet (DFT4FTT) [7], a self-reconfigurable infrastructure for implementing highly reliable ADES. This infrastructure defines a set of hardware and software elements that support the execution of tasks and the transmission of their associated messages while guaranteeing the real-time and reliability requirements imposed on the system.

With regard to hardware, DFT4FTT specifies a system architecture in which nodes, sensors, and actuators are interconnected through a switched Ethernet network. Regarding the software, DFT4FTT follows a centralized architecture in which a software component placed inside the switch, called *Node Manager* (NM), controls the operation of the nodes. Note that both the NM and the switch are duplicated to be able to tolerate their faults, as will be discussed later. The goal of the NM is twofold. During normal operation, it orchestrates the execution of tasks and the transmission of their associated messages. When a relevant change in the operational context occurs, it carries out a self-reconfiguration process. This process consists of determining when the current system configuration does not meet the operational requirements (by a change in either the operational requirements or operational conditions) and, in response, finding and applying a new one that does. Finally, to attain high reliability, DFT4FTT includes various static and dynamic fault-tolerance mechanisms, the last ones relying on the self-reconfiguration process previously introduced.

The rest of the paper is organized as follows. First, in Section 2, we survey some solutions to implement ADES and point out their limitations. After that, we introduce the basic aspects of DFT4FTT. In Section 3, we explain the software model, that is, how the functionalities of the system are represented, and in Section 4, we describe the architecture of the system, that is, the hardware components conforming to the system, how they are interconnected, and the fundamentals of their operation. We then tackle fault tolerance. While in Section 5, we discuss the fault model we assume on the hardware components and the failure semantics we enforce on them, in Section 6, we describe the fault-tolerance mechanisms we designed. In Section 7, we explain the self-reconfiguration process, which is what gives DFT4FTT its ability to implement DFT mechanisms. The feasibility of the proposed solution is demonstrated in Section 8 by presenting its implementation and showing its operation in different operational contexts. We identify the most relevant limitations of DFT4FTT in Section 9. Finally, we summarize the contribution and point out future work in Section 10.

## 2. Related Work

Over the last decades, multiple solutions for building a real-time dependable task-based distributed embedded system have been proposed. We previously conducted a thorough study of these types of solutions in [8], where we classified and characterized them. In that work, we discussed that each of these solutions was designed for a different purpose and, thus, that they are very heterogeneous in the set of services they provide. Note, however, that this depends on the requirements imposed, which in turn have changed over the years. Therefore, we classify these solutions into *classical* and *contemporary*.

The classical class comprises all those solutions that were developed between the 1970s and 2000s. These solutions tended to be small, not very complex, and isolated. Let us explain these properties in more detail. First, the systems were made up of a few interconnected nodes. Second, the operation they had to carry out was typically simple and, thus, the software and hardware required were not very complex. Finally, such systems did not have to coordinate with other systems and, thus, the networks and communication protocols used were specialized. Some of the most relevant projects addressing the development of such solutions are the Software Implemented Fault Tolerance (SIFT) computer [9], the Maintainable Real-Time System (MARS) [10], the Delta-4 [11] or the Generic Upgradable Architecture for Real-time Dependable Systems (GUARDS) [12].

The contemporary class comprises all those solutions that were developed more or less from the 2000s until now. Note, in this regard, that at the end of the 1990s there was a reduction in the price of hardware components which made it possible to develop bigger and more sophisticated DES. This resulted in users demanding more complex properties and functionalities. Some examples are more processing power, more network bandwidth, generality in the design, integration with other systems, and more adaptivity. Some of the most relevant projects that address the development of such solutions are the Generic Embedded System (GENESYS) [13], the Industrial Exploitation of the GENESYS Cross-Domain Architecture (INDEXYS) [14], the Embedded Multi-Core Systems for Mixed-Criticality Applications in Dynamic and Changeable Real-Time Environments (EMC^2^) [15] the Distributed Real-time Architecture for Mixed-Criticality Systems (DREAMS) [16].

It is important to note that although the solutions residing in the second class are more modern, they do not invalidate the ones in the first class. First, from a dependability perspective, note that the basic fault tolerance principles have not changed in decades and, thus, the level of fault tolerance that can be achieved in any of the two classes of solutions is the same. Second, each solution is designed having a set of objectives in mind, and thus, each one is specialized in a given domain or context. In this regard, a classical solution may be more suitable than any contemporary one, depending on the requirements.

Note also that this classification is not strict in the sense that classical solutions can implement, to some extent, functionalities that are more typical for a contemporary solution. In this regard, GUARDS can represent the point of transition between the two classes. This is because GUARDS provide some level of generality as it allows one to create diverse instances of itself to attain different levels of fault tolerance. Another example is its ability to support COTS components that can be upgraded over time.

In general, each of these solutions has its own approach with regard to dependability. Some of them propose specific fault tolerance mechanisms, some others provide different fault tolerance profiles to choose from depending on the application, and some others are very open in the sense that only some guidelines are given. Nevertheless, all of them support active task replication with voting to tolerate affecting the nodes. Moreover, in some cases, they include reconfiguration capabilities to implement additional fault tolerance mechanisms, for instance, to recover faulty task replicas to prevent the attrition of redundancy (see Section 6). However, none of them truly exploits these capabilities to implement dynamic fault tolerance mechanisms, in conjunction with static ones, to provide high reliability. That is why, to the best knowledge of the authors, there is no infrastructure for implementing reconfigurable real-time dependable distributed embedded systems that includes dynamic fault tolerance.

## 3. System Software Model

The operation of a DES is defined by the objectives it has to accomplish, that is, its operational requirements. Among them, the functional requirements are fulfilled due to the execution of *functionalities*. Some examples of functionalities in an airplane are thrust control, climate control, or infotainment. In Section 3.1, we explain how we model the functionalities of the system, that is, we describe how tasks are characterized.

Note, however, that the interval of time during which a system has to operate, or *mission time*, can impose different functional and nonfunctional requirements at different instants if the said system operates under dynamic operational contexts. This can happen due to two reasons. On the one hand, a mission is commonly divided into various *phases*, which are known at design time. Each of these phases defines the set of sub-objectives to be met and, thus, the specific operational requirements to be met at every instant. For instance, in an airplane, the objectives during the taxi phase are different from the ones during the take-off phase. On the other hand, unpredictable events can also lead to changes in operational requirements. For instance, in the event of a catastrophic hardware malfunction, an airplane may require an immediate emergency landing.

Consequently, the normal situation is that the system is providing a subset of all its possible functionalities, each of them with specific non-functional requirements. That is, at every instant of time, some functionalities have to be instantiated with some particular real-time and reliability requirements. In Section 3.2 we explain how we represent the current operational requirements, what we call the *system requirements*. Finally, in Section 3.3 we define in more detail the concept of *system configuration*, that is, how the system can be set up to fulfill the system requirements at each instant.

### 3.1. Modeling Functionalities

As introduced previously, functionalities represent what a system has to do. In DFT4FTT each functionality is implemented by an *application* which, in turn, is composed of a set of interrelated *tasks* that are executed in a sequential and/or parallel manner. More specifically, an application can be represented as a directed graph in which vertices are the tasks and edges are the messages these tasks send among them.

**Figure 2:**
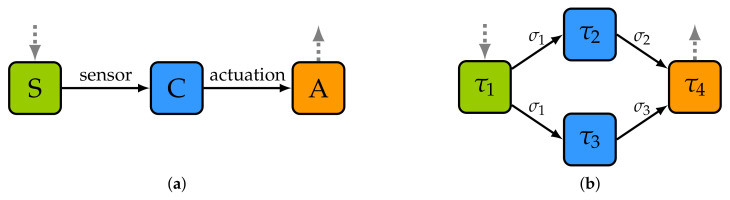
Example of applications. (**a**) Control application example. (**b**) Generic application example.

As an example, in Figure 2a, we depict a basic sequential control application composed of three tasks and two messages. First, task *S* (sensing) consults the value of a sensor. The value captured is then passed to task *C* (control) which determines the actuation value by means of a PID controller algorithm, for instance. Finally, task *A* (actuation) receives this value and performs the corresponding actuation. Note, in this regard, that the execution of an application is a flow of data that starts with at least one input, is processed by multiple tasks, and that finishes with at least one output. Moreover, although there could be several inputs at the beginning of the flow and several outputs at the end, there could also be inputs and/or outputs in the middle of the flow.

Another example, now of a generic application composed of four tasks ($\tau_{1}$, $\tau_{2}$, $\tau_{3}$ and $\tau_{4}$) and three messages ($\sigma_{1}$, $\sigma_{2}$ and $\sigma_{3}$), is shown in Figure 2b. The first task to be executed is $\tau_{1}$. This task is responsible for reading a sensor and sending the sampling data to tasks $\tau_{2}$ and $\tau_{3}$ by means of message $\sigma_{1}$. Bear in mind that an application always has at least one task connected to an external input, like a sensor or a human interface. Additionally, note that, although in this figure two edges emerge from $\tau_{1}$, both correspond to the same message. This task only performs one transmission and the network is responsible for generating two copies of the message and delivering them to the appropriated tasks. After that, tasks $\tau_{2}$ and $\tau_{3}$ process the sampled data in parallel. This results in said tasks sending messages $\sigma_{2}$ and $\sigma_{3}$, respectively, to task $\tau_{4}$. Finally, task $\tau_{4}$ processes the input values and performs some actuation over an actuator. Similarly, as with $\tau_{1}$, every application has at least one task connected to an external output, like an actuator or a human interface.

A more detailed definition of an application can be found in Equations (1)–(3). First, as shown in Equation (1), an application can be represented as a 3-tuple, where Γ denotes its set of *n* tasks, Ψ denotes its set of *m* messages and app_class identifies the application as periodic, sporadic or aperiodic. The different classes of application and their associated attributes are further discussed later in Section 3.2. Second, as seen in Equation (2), each task $\tau_{i}$ can produce a message ($\sigma_{i}$ ∈ Ψ) and can consume various (a set of) messages ($\Psi_{i}$ ⊂ Ψ). Note, however, that there can be tasks that do not produce any message ($\sigma_{i}$ = ∅) and tasks that do not consume any message ($\Psi_{i}$ = ∅). It is important to clarify that, although the symbol ∅ is typically used to represent an empty set, in this case, we also use it to represent when this attribute does not have a value. Third, according to Equation (3), each message $\sigma_{j}$ is produced by one task ($\tau_{j}$ ∈ Γ) but can be consumed by more than one task ($\Gamma_{j}$ ⊂ Γ). Note, in this case, that a message always has one transmitter and, at least, one receiver ($\tau_{j}$ ≠ ∅ ∧ $\Gamma_{j}$ ≠ ∅, ∀*j* = 1…m). Finally, note that the attributes $\tau_{j}$ and $\Gamma_{j}$ represented in Equation (3) contain redundant information, as in Equation (2) attributes $\sigma_{i}$ and $\Psi_{i}$ already define the relations between tasks and messages. Moreover, formally speaking this could lead to a non-valid application definition. For instance, one could state in Equation (2) that task $\tau_{a}$ produces message $\sigma_{b}$ and in Equation (3) that message $\sigma_{b}$ is produced by task $\tau_{c}$, which is an inconsistency. However, for completeness and clarity, we prefer to describe this model this way and we assume that these inconsistencies are not going to occur.

(1) $$\begin{array}{cc} \begin{array}{c} \mathrm{app}=(\Gamma ,\Psi ,app\_class) \end{array} & \end{array}$$

(2) $$\begin{array}{cc} \begin{array}{c} \Gamma =\{\tau_{i}\left(C_{i},\sigma_{i},\Psi_{i}\right),\sigma_{i}\;\in \;\Psi \cup \left\{\emptyset \right\},\Psi_{i}\;\subset \;\Psi ,i=1\ldots n\} \end{array} & \end{array}$$

(3) $$\begin{array}{cc} \begin{array}{c} \Psi =\{\sigma_{j}\left(C_{j},\tau_{j},\Gamma_{j}\right),\tau_{j}\;\in \;\Gamma ,\Gamma_{j}\;\subset \;\Gamma ,j=1\ldots m\} \end{array} & \end{array}$$

Γ: List of tasks

*n*: Number of tasks

$C_{i}$: Worst case exec. time

$\sigma_{i}$: Message produced

$\Psi_{i}$: Messages consumed

Ψ: List of messages

*m*: Number of messages

$C_{j}$: Worst case tx time

$\tau_{j}$: Task producing

$\Gamma_{j}$: Tasks consuming

### 3.2. System Requirements

As already discussed, the operational requirements of the system, both functional and non-functional, can change at runtime. To maintain the specific operational requirements in the current instant, DFT4FTT uses the so-called *system requirements* list. This list contains, from all the applications that could run on the system, only the subset of applications that are required to meet the functional requirements. Moreover, to represent the nonfunctional requirements, each of these application definitions is complemented with their specific real-time and reliability requirements.

Equation (4) depicts one entry of the system requirements. For each application that is required to be executed we specify not only its list of tasks, list of messages, and class but also the attributes related to the real-time (rt_reqs) and reliability (rb_reqs) requirements.

(4) $$\begin{array}{cc} \begin{array}{c} \mathrm{app}\_\mathrm{req}=(\Gamma ,\Psi ,app\_class,rt\_reqs,rb\_reqs) \end{array} & \end{array}$$

The specific list of attributes that is necessary depends on the class of application, as well as on the class of real-time and reliability requirements, at the current instant:

- **Class of application**. As previously introduced, this is a static attribute of the application that indicates when its execution can be activated. A *periodic* application is repeatedly activated after a certain time interval called *period*. A *sporadic* application can be activated at any time, but there is a minimum interval of time between two consecutive executions known as *minimum inter-arrival time*. Finally, an *aperiodic* application can be activated at any time.
- **Class of real-time requirements**. In DFT4FTT, an application can have *hard-*, *soft-*, and *non-real-time* requirements. A hard-real-time application has to finish its execution before some deadline. The result of the execution of the said application is no longer valid after the deadline and this can provoke a system failure, which can have severe consequences. In a soft-real-time application, it is desirable to finish its execution before some deadline. That is, missing some deadlines is allowed but the validity of the result of said execution decreases after the deadline. Finally, a non-real-time application does not have time restrictions in its execution.
- **Class of reliability requirements**. In DFT4FTT, an application can have or does not have reliability requirements. An application with reliability requirements or critical, is one whose operation is indispensable for the correct operation of the system and, thus, if it fails the complete system can fail. That is why this type of application is expected to operate without failing during mission time. As introduced previously, this property is called reliability and its metric is the reliability level, the probability with which, in this case, the application is expected to operate correctly and continuously during the period of time that it is needed. In contrast, an application with no reliability requirements, or non-critical, is one that could fail without impairing the ability of the system to operate in a correct manner. Note, in this regard, that the term critical can be used to refer to highly-safe systems as well as to denote a highly-reliable system. In this paper, we will use this term with the second meaning.

These classifications of application attributes and requirements result in many combinations. However, DFT4FTT only accepts the combinations that we consider more relevant. As shown in Equations (5)–(7) periodic application can be hard-, soft-, and non-real-time. All these three classes of applications require a new attribute *T*, which represents the period. Moreover, hard- and soft-real-time applications have an associated deadline *D*. As concerns the reliability requirements, only periodic hard-real-time applications can be critical. Note, in this regard, that is very difficult to find a critical application that does not require a real-time response. Similarly, it is hard to think of a periodic soft- and non-real-time application that is indispensable for the correct operation of the system. Therefore, we added the reliability level of the application, or rb, only in Equation (5). This attribute represents the probability with which the application must operate in an uninterrupted manner during mission time.

(5) $$\begin{array}{cc} \begin{array}{c} \mathrm{app}\_\mathrm{req}\_\mathrm{hrt}\_p=(\Gamma ,\Psi ,app\_class,(T,D),rb) \end{array} & \end{array}$$

(6) $$\begin{array}{cc} \begin{array}{c} \mathrm{app}\_\mathrm{req}\_\mathrm{srt}\_p=(\Gamma ,\Psi ,app\_class,(T,D)) \end{array} & \end{array}$$

(7) $$\begin{array}{cc} \begin{array}{c} \mathrm{app}\_\mathrm{req}\_\mathrm{nrt}\_p=(\Gamma ,\Psi ,app\_class,(T)) \end{array} & \end{array}$$

As seen in Equations (8) and (9), DFT4FTT also supports sporadic hard-real-time critical and sporadic soft-real-time non-critical applications. From a real-time perspective, in both cases, the minimum inter-arrival time is represented as mit in the expressions below. Additionally, just like periodic applications, sporadic applications have a deadline *D*. From the reliability perspective, just like periodic applications, we do not consider critical applications that do not have strict real-time constraints. Finally, note that we neither consider sporadic non-real-time applications.

(8) $$\begin{array}{cc} \begin{array}{c} \mathrm{app}\_\mathrm{req}\_\mathrm{hrt}\_s=(\Gamma ,\Psi ,app\_class,(mit,D),rb) \end{array} & \end{array}$$

(9) $$\begin{array}{cc} \begin{array}{c} \mathrm{app}\_\mathrm{req}\_\mathrm{srt}\_s=(\Gamma ,\Psi ,app\_class,(mit,D)) \end{array} & \end{array}$$

Equation (10) shows the attributes of the aperiodic applications supported by DFT4FTT. Note, in this regard, only non-real-time and noncritical are considered. On the one hand, as concerns the real-time requirements, aperiodic applications can activate at any instant, which means that the system does not know when they will be executed and, thus, that the scheduler cannot schedule them. On the other hand, regarding the reliability requirements, just like periodic and sporadic applications, we do not consider critical applications that do not have strict real-time constraints.

(10) $$\begin{array}{cc} \begin{array}{c} \mathrm{app}\_\mathrm{req}\_\mathrm{nrt}\_a=(\Gamma ,\Psi ) \end{array} & \end{array}$$

### 3.3. System Configuration

As introduced previously, a change in the operational context can provoke that the operational requirements are no longer met and when this happens, DFT4FTT finds a new proper system configuration that allows it to meet them. A system configuration (or configuration for short) is a given allocation of tasks and messages to nodes and network links, respectively, together with their execution and communication attributes. Note that the specific execution and communication attributes depend on the class of the application and its real-time and reliability requirements.

**Figure 3:**
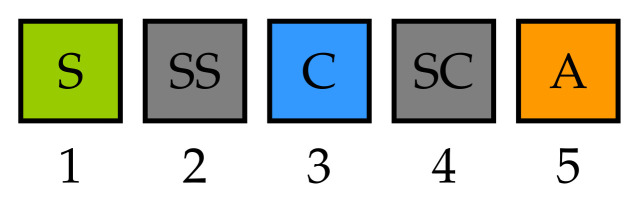
Execution phases of the example control application.

As concerns the real-time response note, first, the execution of an application can be seen as a sequence of task executions and message transmissions. For instance, as shown in Figure 3, the execution of the already mentioned example control application would be composed of 5 execution phases: (1) execution of the task *S*, (2) transmission of data from *S* to *C* (SS send sensor value), (3) execution of task *C*, (4) transmission of data from *C* to *A* (SC send control value), and (5) execution of task *A*. One of the functions of DFT4FTT is to trigger the execution of the tasks and the transmission of the messages in the appropriate instant so that the dependencies between tasks and messages are met and all the deadlines of the applications are fulfilled. For tasks and messages belonging to periodic or sporadic applications, these activation instants are determined by a holistic real-time scheduler (see Section 7.2). For tasks and messages belonging to aperiodic applications, the activation instant is not pre-defined. Instead, the scheduler reserves part of the computing and communication resources for the execution of this type of application.

From the perspective of reliability, as will be explained in Section 6, one of the main fault-tolerance mechanisms in DFT4FTT consists in using task and message replication. In this regard, as will be further discussed in Section 7.2, DFT4FTT also includes a reliability analyzer that determines the appropriate number of task and message replicas to meet the reliability level requirements of the critical applications.

## 4. System Architecture

**Figure 4:**
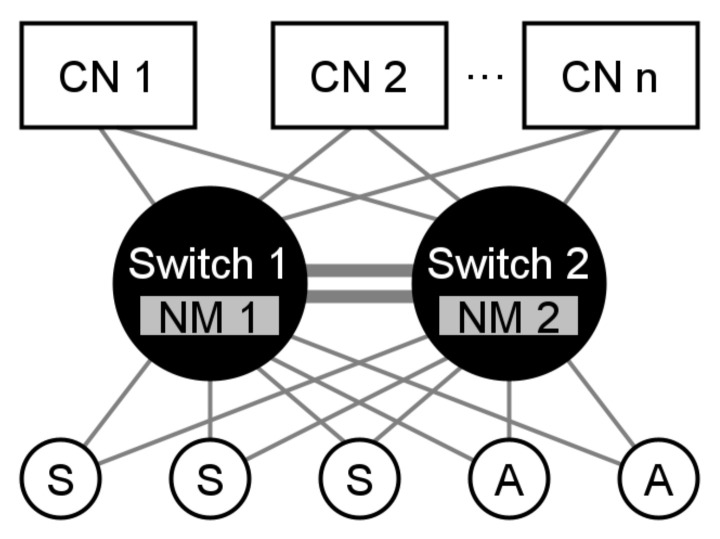
System architecture.

The architecture of the DFT4FTT infrastructure, as can be seen in Figure 4, is composed of several *Computational Nodes* (CNs), *Sensors* (S), and *Actuators* (A) that are interconnected by means of two custom Ethernet switch replicas, each one embedding a *Node Manager* (NM) replica. Next, we describe all these components in more detail.

The CNs are the nodes of the DES, that is, they are the components that execute the tasks. However, CNs do not decide which tasks they execute and when. As explained below, it is the Node Manager (NM) the one that dynamically determines the allocation of tasks in the CNs and then triggers the execution of said tasks and the transmission of their associated messages appropriately to meet the operational requirements.

The sensors and the actuators (SAs) are the components responsible for interacting with the environment. Note that, unlike many DES where SAs are attached to the nodes, in DFT4FTT the SAs are connected directly to the network. This makes it easier for the NM to allocate the tasks in the CNs, as the SAs can be accessed from any CN. Moreover, this makes the architecture more fault-tolerant, as SAs and CNs are spatially separated and, thus, it is hardly possible that they exhibit common-mode failures.

The communication subsystem of DFT4FTT is based on the Flexible Time-Triggered Replicated Star (FTTRS) [17], which is a switched-Ethernet implementation of the Flexible Time-Triggered (FTT) [18] communication paradigm. FTTRS was designed and developed by our group in a previous study and it makes it possible for the nodes of a DES to exchange real-time messages in a flexible and reliable manner. On the one hand, the real-time and flexibility features are provided by the FTT paradigm. However, DFT4FTT does not use the original FTT. Instead, it takes some of the services provided by FTT and adapts or extends them. How this paradigm is implemented in DFT4FTT is further described in Section 4.1. On the other hand, FTTRS introduces several fault-tolerance mechanisms to achieve high reliability in the network. As can be seen in Figure 4, one of them consists in duplicating the communication channel to tolerate faults affecting the physical components of the network. We cover all these mechanisms in more detail in Section 6.1.

Finally, as already introduced, the Node Manager (NM) is a central component responsible for controlling the operation of the rest of the components. As this is an indispensable component, just like the switch, it is duplicated. Actually, as shown in Figure 4, one NM replica is embedded in each of the switch replicas. This physical placement has many advantages in terms of monitoring and configuration. The purpose of the NM is twofold. On the one hand, it is primarily responsible for deciding on the system configuration, that is, for deciding on the allocation of tasks and messages into CNs and links, respectively, and on their execution and communication attributes. Note, however, that this is done dynamically as the operational context changes. For this, the NM carries out a *self-reconfiguration process*, further discussed in Section 7, in which the NM constantly checks if the current system configuration meets the system requirements and, if not, finds and applies a new valid configuration. On the other hand, once a configuration is deployed into the system, the NM is also responsible for triggering the execution of the tasks, and the transmission of their associated messages, so that all the real-time requirements are met.

### 4.1. FTT in DFT4FTT

The Flexible Time-Triggered (FTT) [18] communication paradigm makes it possible for the nodes of a DES to exchange real-time messages in a flexible manner. With flexibility, we mean that FTT provides *real-time flexibility*, as it supports periodic and aperiodic traffic with different real-time requirements and *operational flexibility* as it allows changing the real-time requirements of the traffic at runtime. Due to its properties, FTT is a suitable solution for implementing the communication subsystem of DFT4FTT. Typically, solutions relying on FTT are built on top of it in a multi-layer fashion. However, this is not a good approach for implementing DFT4FTT for three main reasons:

- First, implementing the additional services required to give support to the execution and reconfiguration of tasks on top of FTT is non-trivial. This is because these services require mechanisms already implemented inside FTT, but that are not exposed. For instance, as will be explained below, FTT sets a periodic time base that specifies when to carry out the different actions necessary to transmit, receive, and reconfigure the messages. In DFT4FTT these actions are also carried out but they are a consequence of task-related actions. That is why task-related actions must also be framed in this time base. Unfortunately, this temporal information is not directly available.
- Second, another drawback of adopting FTT as an independent layer is that introduces an overhead. On the one hand, note that in layered architectures there is the added overhead of going through the layers that are not present if components are called directly. This is an important aspect to consider if we want the system to operate in real-time. On the other hand, as will be explained in the next item of the list, DFT4FTT has a different approach than FTT concerning the way a reconfiguration is proposed. In this sense, some reconfiguration-related services provided by FTT are not used in DFTT4FTT. This resource overhead could be removed if FTT is not taken as it is.
- Third, recall from the description of the NM that DFT4FTT carries out a self-reconfiguration process to be able to change the configuration of the system in a semi-automatically manner. This process requires, among other things, recovery data on the operation of the communication subsystem to diagnose it and the ability to reconfigure some of its low-level aspects. Again, this is not possible if FTT is taken as an independent layer.

For all of these reasons, DFT4FTT has been designed to perform a holistic management of tasks and messages. In this regard, DFT4FTT takes the relevant services provided by FTT and adapts/extends them to take into account the dependencies among tasks of the same application and tasks with messages. Moreover, new services have been implemented to carry out the self-reconfiguration process. In this section, we discuss the most relevant FTT-related aspects that have been revised, adapted, and implemented in DFT4FTT.

DFT4FTT divides time into fixed-duration time slots called *Elementary Cycles* (ECs). Every EC starts with the NM sending a so-called *Trigger Message* (TM). The purpose of this message is twofold. On the one hand, it is used to notify CNs, sensors, and actuators when a new EC starts, that is, it is used as a synchronization mechanism. On the other hand, this message contains the EC schedule, that is, it contains the list of tasks and messages that have to be executed and transmitted, respectively, during the current EC. Remember, in this regard, that the NM includes a holistic real-time scheduler for periodic and sporadic applications. In contrast, the activation of tasks and messages belonging to aperiodic applications is not triggered by the TM. They are activated asynchronously.

Regarding the reconfiguration of tasks and messages, DFT4FTT introduces a set of control messages that allow CNs to manually modify the system requirements (see Section 7.2.1). Specifically, these messages allow to request for the execution of a new application, removal of an existing application or change its non-functional requirements. Furthermore, when the configuration of the system has to be changed, either by manual or automatic changes in the system requirements, the NM uses command messages to instruct the CNs to perform low-level changes in the tasks or messages (see Section 7.3).

## 5. Fault Model and Failure Semantics

Following the set of guidelines defined by Avižienis in [19] for building dependable systems, this section discusses the most relevant aspects related to the design and the fault tolerance mechanisms of DFT4FTT. On the one hand, we describe the *fault model*, that is, the different classes of faults that are expected to affect the system. On the other hand, we describe the *failure semantics* we assume on the components of the DFT4FTT architecture, that is, in which manner are they expected to behave when experiencing a failure and how we enforce these failure semantics.

Note that, among the different aspects to be covered according to Avižienis guidelines, we already addressed partitioning of the system in Section 4 by defining the set of software and hardware components conforming to the system, as well as their interconnections. Moreover, the error detection and fault diagnosis mechanisms are later covered when describing the monitoring process in Section 7.1.

### 5.1. Fault Model

The *fault model* describes the types of faults that can affect a given system and the probability of occurrence of said faults. Following the taxonomy defined by Avižienis et al. [20], the fault model of DFT4FTT includes non-malicious operational faults affecting the hardware, both internal or external, natural or human-made, deliberate or non-deliberate, due to accidents or incompetence, and permanent or transient. Two examples of faults that we consider that could affect a DFT4FTT-based system are the deterioration of the physical components and electromagnetic interference affecting the execution of the tasks in the nodes or the transmission of messages in the links. The fault model excludes development, software, and malicious faults. For instance, manufacturing defects, bugs, and intrusion attempts are excluded. As concerns the probability of occurrence of faults, we do not make any assumptions. The only assumptions that we do make are that transient faults affecting links shall be detectable by the Frame Check Sequence (FCS) error detection mechanism of Ethernet and, to be able to parametrize the communication subsystem properly, these transient faults have a known maximum duration.

### 5.2. Failure Semantics

The failure semantics, or failure mode, is the manner in which a system can behave when experiencing a failure. It is important to characterize the failure modes of the subsystems of the system during the design stage as they directly determine the necessary fault tolerance mechanisms. Note, in this regard, that a subsystem potentially failing in an unrestricted manner requires more complicated fault tolerance mechanisms. We now discuss what are the assumptions on the failure modes of the DFT4FTT components (see Section 4) and how we enforce these behaviors to ease the design of the fault tolerance mechanism. For this, we follow the hierarchy of failure modes we proposed in [21], which is strongly based on the one proposed by Poledna in [22].

By default, we assume that any hardware component can fail in an unrestricted manner as we are using Commercial Off-The-Shelf (COTS) components. However, in the case of the switch replicas, which include the NMs, we restrict the failure mode to *crash failures*, that is, they can only fail by omitting to deliver any result for the requested service, and for all the subsequent ones. This is done by using internal duplication with comparison in each switch replica. As concerns CNs and SAs, their failure mode is restricted to *incorrect computation*, that is, they can only fail by delivering incorrect results, either in the value or in the time domain. This is enforced by installing in the switch replicas one Port Guardian (PG) in each link connected with a CN or SA. PGs police the traffic generated by CNs and SAs and discard messages considered invalid. Specific unwanted behaviors eliminated are two-faced behaviors and impersonations [17,23].

## 6. Fault Tolerance Mechanisms

**Figure 5:**
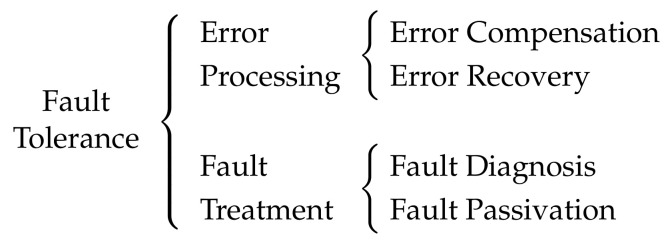
Definition of fault tolerance.

Fault tolerance can be achieved by means of *error processing* and *fault treatment* [24], see Figure 5. Error processing aims at removing errors from the state of the system before they provoke a failure. This can be carried out by means of two different techniques. On the one hand, *error compensation* consists in providing enough redundancy so that the system produces correct results, even in the presence of faults. Some types of redundancy [25] that could be used in a DES are hardware (or space) redundancy, which involves providing additional hardware components, like nodes, links, switches, or sensors; software redundancy, which involves providing additional software, like redundant tasks; and time redundancy, which involves performing the same action multiple times, with the same hardware and software, like message retransmission. On the other hand, *error recovery* consists in, first, identifying the system state as erroneous and then replacing it with an error-free state. Note that, in the first step, *error detection* mechanisms are used. Actually, the term *error detection and recovery* is usually utilized. Furthermore, if the error-free state is a previous state, the technique is called *backward recovery*; whereas if it is a new state, it is called *forward recovery*. On the other hand, fault treatment aims to prevent faults from provoking errors again, which is a two-step process. First, *fault diagnosis* is carried out to identify the fault that causes errors. Second, *fault passivation* is used to prevent the activation of the fault again. This can be done by disabling the faulty subsystem.

To attain high reliability in a real-time system, it is necessary that the errors do not impair the ability of the system to meet its deadlines. This is more achievable if errors are tolerated without introducing a recovery time. That is why DFT4FTT primarily utilizes error processing with error compensation. For example, as will be explained in Section 6.1, critical tasks and the messages they produce are replicated. Moreover, task replicas periodically perform a majority voting on their results so that errors affecting them can be tolerated even without being detected. This is called *fault masking*.

Note, however, that error compensation may not be enough to provide the required level of reliability in critical systems. This is because faults affecting the task replicas, even if the system is able to tolerate them, may decrease the available level of redundancy. This issue is called *redundancy attrition* and reduces the ability of the system to tolerate additional faults. It is important to address redundancy attrition as it can be caused not only by permanent faults but also by temporary faults, which are more likely to occur than permanent ones. DFT4FTT prevents redundancy attrition using a combination of complex techniques based on error recovery that allows for salvaging faulty replicas of tasks. We define various techniques, each one addressing a different level of fault severity. The first technique is a classical forward error recovery mechanism in which a faulty replica uses the result of the vote to correct its own contribution (see Section 6.2). For faults that cannot be dealt with using forward error recovery, DFT4FTT includes two *reintegration* techniques based on a previous study [26] (see Section 6.3 and Section 6.4). Reintegration is similar to error recovery, but the new error-free state is obtained as a result of reaching an agreement and performing the necessary resynchronization with other components of the system [21], in this case, with the other replicas of the group. If the previous techniques do not suffice, a *restoration* of the replicas is carried out. This is a technique we developed in this work and takes advantage of the reconfiguration capabilities of DFT4FTT to reallocate the faulty replica to a different CN. After that, the previous techniques are used to reintegrate the new replica with the other replicas of the group (see Section 6.5). Finally, note that some of these techniques can also be used with non-replicated tasks. In this case, the service provided by those tasks is lost until these techniques manage to regain said service.

The reconfiguration capabilities of DFT4FTT are not only used to prevent redundancy attrition. Apart from that, they make it possible for the NM to dynamically select the number of replicas for tasks and messages, depending on the operational context, to consume as few resources as possible while guaranteeing the required level of reliability. That is, as explained in Section 6.6, we can reconfigure the redundancy. Furthermore, in Section 6.7 we discuss a technique that has not been implemented in this work due to its complexity but that fully exploits the reconfiguration capabilities of DFT4FTT to maximize the reliability of the system while minimizing the amount of resources required. The idea is to reconfigure not only the number of replicas for tasks and messages but to be able to select the appropriate fault tolerance strategy at every instant.

In the rest of the section, we describe the set of fault tolerance techniques contained in DFT4FTT. As shown in Table 1, we divide these techniques into static, here we find both classical and advanced techniques; and dynamic, the ones that make use of the reconfiguration capabilities of DFT4FTT. Each technique can include several associated fault tolerance mechanisms. In the last column, we identify each mechanism as reused (R) if no significant modifications were necessary to integrate it; adapted (A) if some modifications were necessary; and new (N) if it was explicitly designed for DFT4FTT.

### 6.1. Error Compensation

As already introduced in Section 4, to attain high reliability at the network level, DFT4FTT relies on the Flexible Time-Triggered Replicated Star (FTTRS) [17]. To tolerate permanent faults affecting the network FTTRS uses space replication in the communications. Specifically, as shown in Figure 4, CNs and SAs communicate between them through two switch replicas, which are interconnected through two *interlinks*, so that the switch replicas can coordinate their operation. Note, additionally, that this configuration also allows one to tolerate temporary faults affecting the network. In particular, the type of temporary network fault that is more relevant due to its probability of occurrence is a temporary fault affecting a link. This type of fault can corrupt the messages being transmitted which, thanks to the CRC error detection mechanism of Ethernet (see Section 7.1.2), results in dropped messages. In any case, since the assumption is that the temporary fault is affecting only one link and each pair of nodes communicate redundantly via both switches, we can assume that messages will correctly reach their destinations via the path which is not affected by the fault, thus tolerating the temporal fault. However, tolerating temporary faults by means of hardware redundancy is not very efficient because, in the event of a permanently faulty link, this fault tolerance is lost. This calls for a more suitable fault-tolerance mechanism.

In many systems temporary faults affecting the messages are tolerated using Automatic Repeat Request (ARQ), that is, the transmitter of a message retransmits said message if the receiver does not acknowledge the reception after a reasonable period of time. ARQ is not the most appropriate approach for real-time systems since it introduces a non-negligible and variable amount of delay. That is why in FTTRS temporary faults affecting the links are tolerated by means of temporal replication. That is, critical messages are proactively retransmitted *k* times, being *k* a static value that is proportional to the expected network fault occurrence ratio. However, as explained in Section 6.6, in DFT4FTT, this value is dynamically adjusted to address changing operational contexts efficiently and effectively.

Regarding the NM, it is duplicated to avoid being a single point of failure. Moreover, the location of the NM replicas is crucial since they must be able to control the operation of the CNs and the SAs. That is why they are placed in the center of the system, one inside each switch. Note, additionally, that the two switch and NM replicas must be *replica determinate* [22], that is, in absence of faults, assuming both replicas start with the same state and receive the same inputs, they will produce the same outputs. This aspect has already been addressed for the switches in [17]. The specific mechanisms ensuring replica determinism between the NM replicas deserve more space than the one we can use here. That is why their description is left for a future document.

**Figure 6:**
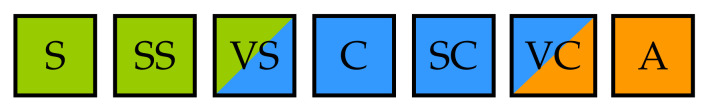
Execution phases of a replicated control application. The colors denote the tasks involved. Green is task *S*, blue is task *C*, and orange is task *A*.

Finally, at the node level, DFT4FTT uses task active replication with a majority voting to tolerate permanent and temporary hardware faults affecting the CNs. More precisely, each critical task is replicated and executed in parallel in different CNs. Additionally, task replicas periodically vote on their results to obtain a consensus result. When, what, and how to vote depends on the application. However, we already proposed, in a previous study [26], an execution scheme for a replicated control application. Specifically, we assume the type of application depicted in Figure 2a, a basic control application composed of three tasks *S*, *C*, and *A*. For each of these tasks, several replicas are created and installed in different CNs. To execute the application while ensuring consensus on replicas, we extend the execution scheme shown in Figure 3 with new phases each one corresponding to one or more ECs. This new execution scheme is shown in Figure 6.

First, in phase Sense (S), replicas of task *S* obtain the value of their corresponding sensors. In phase Send Sensor (SS) each of these replicas sends its obtained value to the other replicas and, also, to the replicas of task *C*. Then, in phase Vote on Sensor (VS), replicas of both tasks *S* and *C* perform a majority voting with the received values. Note, in this regard, that replicas of task *S* use the result of the vote for diagnosis (see Section 6.4), while replicas of task *C* use it as their input value. During phase Control (C), replicas of task *C* execute the control algorithm to obtain the actuation value, using the consensus sensor value previously received. After that, the approach followed in phases SS and VS is repeated. On the one hand, in phase Send Control (SC), each replica of task *C* sends the result of the algorithm to the other replicas and, also, to the replicas of task *A*. On the other hand, in phase Vote on Control (VC), replicas of both tasks *C* and *A* carry out a majority voting on the actuation values. Finally, in phase Actuate (A), each replica of task *A* performs the actuation previously obtained from the voting, using the corresponding actuators. Note that, when actuators are replicated, some voting mechanism has to be implemented at the physical level [12] so that, if an actuator replica uses an erroneous value, the remaining ones are capable of setting the controlled device in the correct state.

Regarding the number of replicas for each task, it has to be odd for the majority voting to work properly. In general, to tolerate *t* faulty CNs, at least 2t+1 task replicas must be used. In particular, critical tasks have 3 or 5 replicas, depending on their reliability level requirements and the operational conditions under which the system operates.

### 6.2. Error Recovery

The voting procedure explained in the previous subsection makes it possible for a task to provide a correct service, as long as a majority of its replicas operate correctly. Note, in this regard that if a fault prevents a task replica from operating correctly, the voting directly masks the error, without the need to detect it. As already indicated, this is called fault masking. However, if a task replica compares its proposed value with the result of the voting, it can detect the error and even correct its internal state by substituting the proposed value with the consensus value. By doing this, we could recover a faulty replica and, thus, prevent redundancy attrition. Actually, this is a classical forward error recovery mechanism and, again, its implementation depends on the application. Specifically, is the developer’s decision when and how this recovery is carried out.

In the type of control application that we assume, the only task that can take advantage of this error recovery mechanism is task *C*. This is because the result of the control algorithm is the only one that can depend on the result obtained by the same algorithm in its previous execution, that is, it has an internal state that determines the next result. Conversely, tasks *S* and *A*, in principle, do not have an internal state, they just forward the value they receive. Consequently, to implement error recovery in this type of application only phase VC must be modified to carry out the actions described above, which is to substitute the proposed value with the consensus value. In case the application is more complex and, thus, there are more phases, the error recovery could be implemented in several of these phases so it occurs more often, which could reduce the amount of time the internal state of a task replica is inconsistent with respect to the other replicas.

Note, however, that some faults may lead a task replica to desynchronize at the communication or application level. These faults require a fault tolerance technique beyond simple error recovery. That is why in the next section we describe two more sophisticated recovery mechanisms we refer to as reintegration mechanisms.

### 6.3. Reintegration of Lost Redundancy

To prevent the redundancy attrition provoked by permanent or temporary faults affecting any of the CNs or the internal state of any of the critical tasks, DFT4FTT includes two levels of reintegration. Here we describe the first level, which is composed of two mechanisms that are designed to help a task keep in synchronization with the global timing and to help the replicas of a task to keep coordination among them.

The first reintegration mechanism is the result of the manner in which CNs activate the tasks they execute. As previously explained in Section 4, the NM is responsible for periodically triggering the activation of tasks and the transmission of their message. Consequently, if a task or a CN suffers from a temporary fault that causes a temporal desynchronization, the system should be able to resynchronize it upon the next triggering.

The second mechanism makes it possible for the task replicas to recover from a temporary fault affecting its internal operational state. Note, in this regard, that most tasks maintain several values that represent their operational state. For instance, the state in a task implementing a PID controller is the previous value of what in control theory is called *error*, that is, the difference between the desired setpoint and the measured process variable. If the state of a task like this is corrupted, it will permanently fail in delivering correct results. This fault tolerance mechanism is an extension of the voting described in the previous sections, and the main idea is to exchange and vote not only on the main output the task replicas produce but also on the operational state of the application. The implementation of this mechanism in the type of control application we assume implies modifying, on the one hand, phase SC so that each replica of task *C* piggybacks its state in the messages sent and, on the other hand, phase VC, so that each replica of task *C* substitutes its own state with the result of the voting on the states of all the replicas.

### 6.4. Reintegration of Very Lost Redundancy

In some cases, a fault can corrupt the internal state of a task replica, or the CN where this replica is being executed, in such a way that it is not possible to reintegrate it using the just-described mechanisms. That is why DFT4FTT implements various fault-diagnosis and reset mechanisms that make it possible for the NM to detect when a task replica is affected by this type of fault and then restart the affected component to remove the error.

The fault-diagnosis mechanisms rely on two types of error counters: the Communication Error Counter (CEC) to diagnose problems in the communications and the Discrepancy Error Counter (DEC) to diagnose problems in the operation of the task replicas. There is one CEC for each message and one DEC for each replicated task, which are kept both in the NM and in the involved CNs. Moreover, the CNs and the NM collaborate to update the values of all of these counters properly. Note that these error counters are not exclusively used in the scope of this fault tolerance technique. The information they provide is taken into account, together with other information, to carry out a system-level diagnosis which is part of the self-reconfiguration process of DFT4FTT. Therefore, the procedure followed to update their value is further explained in Section 7.1.2.

If the value of any of these error counters surpasses a pre-defined threshold, the affected task, the affected CN, and the NM try to recover the lost resources by carrying out the next sequence of three actions. This procedure finishes when the error is successfully removed or after the last action is carried out.

First, the task replica tries to reset itself to remove the error. The software reset consists in initializing the internal state and starting the execution of the task replica from the beginning. Second, if the NM determines that the task replica was unable to remove the error, for instance, because the fault affected the ability of the task replica to update the error counters properly, it sends a command message instructing the task replica to reset. Finally, if the software reset did not suffice to remove the error, the NM sends a command message instructing the CN to reset. When this occurs, the CN initializes its internal state and then starts the execution of all the tasks assigned to it.

Note, at this point, that a node may fail to perform a hardware reset when instructed. To overcome this issue, we propose a fault tolerance mechanism based on a watchdog timer (WDT). Specifically, the NM periodically sends a You Are Alive message (YAA) to every CN considered non-faulty. Then, each CN forwards this YAA message to a dedicated WDT, which is directly attached but is independent of it. This WDT is responsible for performing a hardware reset when several YAA messages have not been received for a certain amount of time. To prevent the WDT from processing a forged YAA message unintentionally generated by the CN, the NM includes in each YAA message a signature that is dynamically updated in a way that only the NM and the WDT know. Note, additionally, that this mechanism can also be useful for recovering a CN when a communication error prevents it from communicating with the switches.

Finally, note that the set of reset mechanisms described here involves initializing the internal state of the task replicas. Consequently, although they succeeded in removing the error, the replica would not be synchronized with the other replicas of the group. That is why the reintegration mechanisms described in the previous section are used afterward.

### 6.5. Restoration of Lost Redundancy

In some cases, the set of fault tolerance mechanisms described in the previous section may not suffice to reintegrate the faulty replica due to the severity of its erroneous situation. One example is a crash of a CN caused by a permanent fault. However, note that the NM can still try to prevent redundancy attrition by instructing the reallocation of the affected tasks to other CNs and then reintegrating them using the mechanisms described in Section 6.3. We call this technique *restoration* and it is only possible thanks to the reconfiguration capabilities provided by the NM.

For critical (replicated) tasks this means that we can implement an N-Modular Redundancy (NMR) with several spares. However, unlike the classical NMR scheme, here we deal with software spares that can be continuously reallocated, as long as there are enough communication and computational resources in the system. Similarly, as with the mechanisms described in the previous section, restoration can also be used with non-replicated tasks, however, in that case, the associated application will not provide its service during the duration of the restoration process.

It is noteworthy that all the fault tolerance techniques that make use of the reconfiguration capabilities of DFT4FTT, like this one and the ones we will explain in the next two subsections, rely on the self-reconfiguration process carried out by the NM. In this regard, as will be explained in Section 7, all reconfiguration decisions are taken in a holistic manner, that is, considering all the fault tolerance mechanisms at the different levels of the architecture. Recall from the introduction that this is necessary if we want the fault tolerance mechanisms to operate in an efficient and effective manner.

### 6.6. Reconfiguration of the Redundancy

The reconfiguration capabilities of DFT4FTT are not only used for implementing the restoring mechanism previously described. This infrastructure also includes mechanisms to dynamically select the number of replicas for tasks and messages, depending on the operational context. Specifically, when the system starts operating in a more stringent operational context, the number of replicas can be increased to maintain the level of reliability. Conversely, the number of replicas can be decreased to free resources when the operational context is more benign.

### 6.7. Reconfiguration of the Fault Tolerance

In the previous sections, we showed how the reconfiguration capabilities of DFT4FTT make it possible to implement advanced fault tolerance mechanisms for reallocating tasks, preventing redundancy attrition, and dynamically selecting the appropriate number of replicas for tasks and messages to maximize reliability while minimizing the consumption of resources. Note, however, that DFT4FTT has been designed having flexibility in mind and, thus, it has a level of reconfigurability that allows implementing even more complex and dynamic mechanisms. In this sense, we have considered the possibility of accommodating other fault tolerance strategies to both improve fault tolerance and give support to other dependability attributes.

On the one hand, recall from Section 6.1 that DFT4FTT uses active task replication with majority voting. This mechanism is adequate for building highly-reliable systems. However, in some scenarios, other approaches could be more suitable. For instance, if the system is operating in a degraded mode, less critical tasks could use duplication with the comparison [25] so that more resources can be used for other more critical tasks. That is, in extreme situations, we could sacrifice reliability in less critical tasks to provide better reliability to more critical tasks. On the other hand, DFT4FTT could include additional fault tolerance strategies to cope with tasks with dependability attributes other than reliability. Note, in this regard, that, as mentioned in the introduction, dependability contains several attributes [1]. For instance, availability is a “measure of the delivery of correct service with respect to the alternation of correct and incorrect service”. That is as availability assumes that the system can sometimes provide an incorrect service, it is less stringent when compared with reliability, which requires the service to be provided continuously. In this sense, DFT4FTT could give support to tasks with availability requirements in an efficient manner if techniques like duplication with comparison or passive replication are implemented [25].

Finally, note that these are preliminary ideas that have not been reflected in the current design of DFT4FTT. Implementing this technique requires adding higher levels of intelligence into the NM to take the decisions and additional components in the NM and the CNs to implement the fault tolerance mechanisms that support the specific strategy.

## 7. Self-Reconfiguration Process

The most important feature of DFT4FTT is its ability to dynamically manage the computation and communication resources so the system can operate correctly, even if its operational context changes. As already explained, this is interesting from a functional perspective, as the system can change the services it provides to meet changing requirements, but also from a dependability perspective, as we can implement more effective and efficient fault tolerance mechanisms (see Section 6). This ability is achieved by means of what we call the *self-reconfiguration process*, in which the NM and CNs collaborate to constantly carry out three consecutive subprocesses called: (1) *monitoring process* to monitor the environment and the system itself to obtain the system state; (2) *decision process* to determine if the system state fulfills the system requirements and, if not, propose a new system configuration that does, and (3) *configuration change process*, to apply the new configuration to the system.

**Figure 7:**
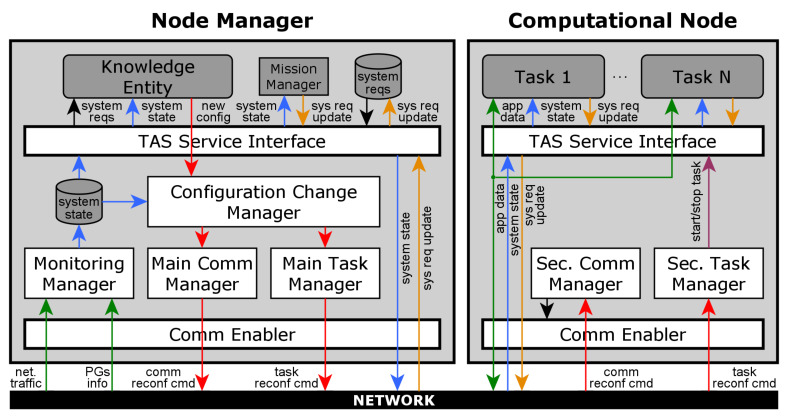
Internals of the Node Manager and a Computational Node.

In this section, we describe each of these subprocesses in detail. For this, we rely on Figure 7 in which we show the internals of the NM, on the left, and one CN, on the right. Specifically, we show the most relevant software components involved in the self-reconfiguration process, their dependencies, and the information they exchange. Note, in this regard, that there are several levels in this architecture. At the bottom, we have the network, which allows the NM and the CNs to exchange application data and control messages. Recall from Section 4 that it is based on FTTRS. Moreover, the *Communication Enabler* component acts as an intermediary between the network and the rest of the system components. In the center of the figure, we can see the low-level modules responsible for collecting and diagnosing the state of the system, as well as for managing changes in the tasks and messages. Above that, the *Task Allocation Scheme (TAS) Service Interface* makes it possible for the set of high-level components to interact with these low-level components in an easy manner. At the top, in the NM, we find the high-level components responsible for instructing the changes in the system configuration when necessary; and, in the CNs, the tasks themselves being executed.

### 7.1. Monitoring Process

The first step in the self-reconfiguration process is collecting the necessary information to determine if a change in the system is needed. Specifically, the NM monitors the behavior of the whole system to obtain a logical representation of its current state called *system state*. The system state, as shown in Figure 7, is obtained by means of the Monitoring Manager (MM). For this, the MM collects information from the PGs (see Section 5.2 and Section 7.1.2), inspects the messages generated by each CN, and then infers the status of each hardware and software component. The reason for inferring the state of the CNs is that we assume they can fail by providing incorrect values and, thus, the information they could provide about their status would not be reliable. Note, additionally, that collecting all this information is possible thanks to the privileged placement of the NM in the architecture.

The system state is constituted by different information collected from several parts of the system. Next, in Section 7.1.1, we explain, for each ingredient of the system state, which data is gathered and how it is processed. Later, in Section 7.1.2, we describe the diagnosing mechanisms that make it possible to identify the faulty components of the system.

#### 7.1.1. System State

As introduced, the system state constitutes a snapshot of the current condition of the system. Specifically, it encompasses different information about the hardware and software:

- **Status of the hardware**. This is the list of hardware components, how they are interconnected among them and whether they are permanently faulty or not. To determine the status of the architecture, the MM is fed with the initial architecture of the system, which is then updated upon the detection of any hardware component suffering any fault considered permanent. Note that, as previously explained, this is done exclusively by inspecting the messages transmitted through the network. Specifically, the MM keeps track of the messages transmitted and detects untimely transmissions and, in particular, omissions. If the behavior of a CN or a SA deviates from its specification for a significant amount of time, it is considered permanently faulty. Further information about the diagnostic capabilities of the NM can be found in Section 7.1.2.
- **Reliability of the hardware**. This is the probability with which CNs, SAs, and links are expected to suffer from faults. Note, in this regard, that this probability can change dynamically depending on the harshness of the environment. In the case of CNs and SAs we are interested in their probability of failing, that is, suffering a permanent fault. For the links, we are interested in their probability of suffering, both permanent and temporary faults. That is, the probability of losing a link and the probability of losing a message in a specific link.
- **Status of the software**. This is the list of tasks executed in each of the CNs and whether these tasks are faulty or not. There are two ways in which the MM determines the correct or faulty state of a task. On the one hand, the MM supervises the traffic generated by each of the tasks to be aware of any message omission. This makes it possible to detect a crash in the task or errors affecting its communication. On the other hand, the MM also performs the voting on messages generated by replicated tasks and then compares the consensus value with the actual value proposed by each of the replicas. This makes it possible to detect errors affecting the internal state of a task replica. Note that, for diagnostic purposes, tasks send their messages to the network, even if the receiving task is in the same node.

#### 7.1.2. Diagnosis

Here we describe in more detail the list of techniques that can be used by the MM to diagnose both temporary and permanent faults affecting the CNs, SAs, and their links. This will help to obtain most of the information contained in the system state. For this purpose DFT4FTT uses two diagnosis techniques: *model-based* and *behaviour-based*.

The model-based technique is used in DFT4FTT to determine the reliability level of a given hardware component when the environment changes. For this, the MM monitors the environmental conditions under which the system is operating. This monitoring can be done by means of the sensors already available in the system and, additionally, by means of new ones installed to capture specific environmental attributes. These data are then used as the input for a specific model of the hardware component. For the CNs, SAs, and links, since we are interested in their probability of failure, a reliability model is used. An example of this kind of model is the one proposed in the MIL-HDBK-217 handbook [27].

The behavior-based technique is used in DFT4FTT to infer the status of a given hardware or software component solely by inspecting its outputs. Next, we list the different sources of information we use and explain how we can process the data they provide to determine the system state.

- **Error counters of the network interfaces**. Each of the two endpoints of a link is connected to a Network Interface Controller (NIC) implementing the Ethernet protocol. NICs include mechanisms for detecting and treating network errors. Moreover, the information regarding the detection of errors is made available in the form of error counters. Some examples of the information that can be consulted are the number of malformed frames, collisions, or CRC errors. The MM uses this information to determine the reliability of the links. On the one hand, it can determine the ratio of occurrence of temporary faults. On the other hand, if the occurrence of temporary faults is so high that the communication in a link cannot succeed, the link and its associated CN or SA are considered permanently faulty and the status of the hardware is updated accordingly.
- **Port Guardians**. As introduced in Section 5.2, behind every NIC of each DFT4FTT switch, there is a module called Port Guardian (PG) [23] that prevents the propagation of errors generated by the CNs and the SAs. Specifically, they discard any incoming or any outgoing message considered incorrect from the DFT4FTT perspective. For instance, they detect and thwart attempts of impersonations and two-faced behaviors [17]. Moreover, PGs have access to the message scheduling and, thus, they can also detect messages sent untimely and messages omitted. This diagnosis information is made available so that the MM can infer the probability with which links suffer temporary faults, as well as identify permanently faulty links, CNs, tasks, and SAs.
- **Acknowledgement messages**. As explained in Section 6.4, DFT4FTT contains multiple error counters that allow one to diagnose problems affecting the CNs. Moreover, there are two copies of each of these error counters, one in the NM and one in the associated CN. One type of error counter is the Communication Error Counter (CEC), which helps to diagnose communication problems. In particular, each CEC increases its value each time its associated CN is not able to transmit or receive a message. While the NM can easily check if a CN is transmitting properly as it keeps track of all the traffic passing through the switches, assessing the reception requires that the CN informs about its received messages. This is done by means of acknowledge messages, which are also used by the MM to determine the reliability level of the links. Specifically, the MM can determine the probability of occurrence of temporary faults affecting the downlinks, as well as identifying permanent faults affecting the links and CNs.
- **Discrepancy Error Counters**. The MM also consults the Discrepancy Error counters (DECs) to have information about the ability of replicated tasks to generate a correct value. Specifically, as explained in Section 6.1, task replicas periodically exchange their partial results and then vote on them to obtain a consensus result. Each time a task replica detects that its proposed result significantly differs from the consensus result, it is considered that it is not able to produce correct results and, thus, its associated DEC is increased. This is most likely to occur due to hardware errors affecting the CN. An example is a bit flip in the memory affecting the internal state of the task. Consequently, the DEC can serve as a means to determine the reliability level of the CNs. Moreover, this diagnosis mechanism is also used by the MM to determine the correct or faulty state of the tasks in the status of the software. Specifically, if a task replica is unable to produce correct results for a significant amount of time, it is considered permanently faulty.
- **Node heartbeat**. Note that all the sources of information previously described relying on the tasks and CNs producing messages. Consequently, the MM can only do this diagnosis if they are operating. Therefore, if the periodicity with which tasks in a CN activate is large, identifying a fault can take a significant amount of time. To overcome this issue DFT4FTT contains a heartbeat diagnosis mechanism consisting of each CN periodically responding to the reception of each TM with an I Am Alive (IAA) message. This makes it possible for the NM to periodically assess the ability of the CNs to receive a message, generate a response message, and transmit this last message back to the NM. Note, however, that a CN could fail in a way that it is able to produce IAA messages but that it is not able to carry out high-level procedures like executing a task. To also assess the ability of the CNs to execute code, the content of each IAA message is the result of executing a function that depends on the content of the TM.

### 7.2. Decision Process

The decision process is responsible for selecting the appropriate system configuration when the system state does not meet the system requirements. The central component in this process is the *Knowledge Entity* (KE). As shown in Figure 7, the KE consults the system state, as well as the system requirements, and outputs a new configuration, if necessary. Specifically, it extracts from the system state the set of applications that are being executed and compares it against the list of tasks from the system requirements to assess the functional requirements. Moreover, the KE also extracts the behavior of the tasks to verify that their real-time and reliability requirements are also fulfilled. If both functional and non-functional requirements are met, the system configuration is left as it is. Otherwise, the KE proposes a new system configuration that fulfills the system requirements.

Next, in Section 7.2.1, we explain how the system requirements (which were first introduced in Section 3.2) are implemented in the NM. We discuss who can modify them and how they should be modified. Later, in Section 7.2.2, we explain how the KE can determine if a new configuration is required. Finally, in Section 7.2.3, we describe how the KE can find a new configuration that fulfills the system requirements. Note that, as stated in the introduction, this search process could be very complicated and time-consuming. This is because the number of potential configurations is huge. Moreover, assessing the real-time and reliability properties of a configuration could also be non-trivial. Actually, this is a search problem, which is a very well-known problem existing in many domains and still an open issue to be solved in an acceptable amount of time. That is why we will not provide a final solution. Instead, we will present the problem, identify the actors involved and point out different potential solutions for finding the new configuration.

#### 7.2.1. System Requirements

We already described the system requirements in Section 3.2. In that section, we explained that it is a list of applications, together with their real-time and reliability requirements, that are necessary to meet the operational requirements of the system. This is a very important piece in the self-reconfiguration process as it dictates the system configuration. In this sense, we must define well who can modify this list and in which manner so that the reconfiguration is not compromised. In this section, we discuss the internal organization of the system requirements, as well as how and by whom it can be modified.

First of all, regarding the internal organization of the system requirements, applications are divided into two groups depending on the event that triggered their need for execution.

The phase-related applications are those indispensable applications needed to fulfill the operational requirements of a given phase of the mission. Each phase starts as a result of the fulfillment of a specific condition. For instance, in a commercial flight, when the plane reaches a certain altitude, it is considered that the *climb* phase has finished and that the *cruise* phase has started. The Mission Manager in the NM is the one responsible for properly updating the system requirements when the phase of the mission changes. For this, before starting the operation, the Mission Manager is fed with the mission specification. Then, at run-time, the Mission Manager inspects the system state, detects whether any phase-related condition is fulfilled, and, if so, updates the system requirements accordingly.

The on-demand-related applications are those either indispensable or non-indispensable applications that are started to cope with unplanned events occurring in the system. These events are the result of either an external command sent by a human or an internal request sent by an application. On the one hand, the users of the system can send commands to change its operation. Some examples are a passenger of a vehicle requesting multimedia services to the application responsible for the infotainment subsystem which then starts an additional application to provide such services; and the driver of the vehicle sending some overruling command, forcing the system to start a critical application to support said command. On the other hand, tasks are also allowed to modify the system requirements. Note, in this regard, that, although DFT4FTT tries to automate as much as possible the operation of the system, designers can create applications that are allowed to change the configuration of the system. This provides designers with full control of the reconfiguration capabilities. Tasks, by means of *system requirement updates*, are allowed to start and stop applications, as well as to modify the real-time and reliability requirements of the already running applications (both phase-related and on-demand). Note that modifying the phase-related applications allows one to do overruling.

These change requests are very powerful as they allow them to completely modify the operation of the system. However, CNs can fail by producing incorrect results, so requests could contain wrong information which would result in a wrong system modification. To solve this issue, we ensure that requests affecting critical applications are only issued in a reliable manner. For this, two different approaches are proposed. On the one hand, critical requests can be issued by specific highly-reliable CNs. On the other hand, when a highly-reliable CN cannot be provided, the task issuing a critical request must be triplicated, and said request must be agreed upon among the three replicas.

#### 7.2.2. Detecting the Need for a New Configuration

The need for a new configuration is triggered by a change in the operational context, which results in a change in the system state or system requirements. When this happens, the KE checks if the current system requirements are met, taking into account the system state. This involves, first, checking the functional requirements. For this, the KE extracts the list of tasks that must be executed from the system requirements and the list of tasks that are being executed from the system state. If there is some discrepancy, the configuration must be changed to introduce the new required tasks or to remove those that are no longer necessary to free the resources.

Secondly, the non-functional requirements must also be checked. As concerns the real-time requirements, there can be discrepancies if the real-time requirements of an application have been changed. To check that the new requirements are met the KE uses a holistic schedulability analyzer, that is one that considers tasks and messages together. We propose to use the work described in [28] to determine a set of execution attributes that ensure that all applications meet their deadlines. Afterward, we propose to use a task schedulability analyzer and a message schedulability analyzer to ensure that, considering these execution attributes, the available computational and communication resources also suffice to meet the deadlines. Additionally, changes in the operational context can provoke reliability requirements to stop being met if the environment changes to a more stringent one or if the reliability requirements change. The assessment of the reliability requirements calls for a reliability analyzer. Specifically, the NM is provided with a parametrized model of the system and checks if the level of reliability of the current system state is equal to or higher than the required reliability level during the mission time. Finally, note that these type of analyzers typically are very time-consuming. This is a problem since, during the self-reconfiguration process, the system is not providing the expected service. Moreover, from a fault tolerance perspective, the longer the system is suffering an error, the more vulnerable it is to the occurrence of additional errors. That is why it is desirable these analyzers are online, that is, they are designed to minimize the response time.

#### 7.2.3. Finding a New Configuration

In case the current configuration of the system does not meet the system requirements, the KE carries out a search to find a proper new one. We have identified three different aspects involved in this search that are mandatory or desirable:

- The search, among all the possible configurations, has to find a valid configuration, that is, one that fulfills both the functional and non-functional requirements.
- The time required to find a new valid configuration must be short enough to ensure that the properties of the system are not compromised. The specific expected behavior depends on the system itself. Moreover, this time has to be bounded if we want that the self-reconfiguration process is carried out in a real-time manner.
- It is desirable that the new configuration is as similar as possible to the current one, that is, it is preferable that that configuration change process involves as few changes as possible. Note that in this regard, as will be explained in Section 7.3, applying a new configuration could be complicated depending on the number of changes.

**Figure 8:**
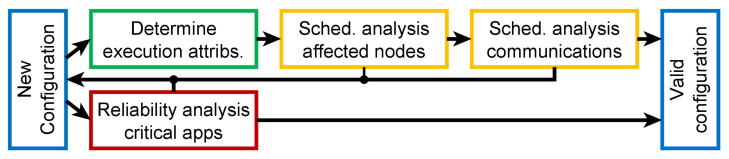
Diagram of the first search approach where the assessment of the real-time and reliability requirements is done separately.

Apart from being the main source of time consumption, introducing non-functional restrictions into the search process is not trivial. That is why we propose two search approaches, each addressing these requirements in a different manner:

1. The first search approach consists in taking the list of tasks that are required to be executed from the system requirements and generating all the combinations of task distributions, that is, generating one by one, all the possible configurations that fulfill the functional requirements. As shown in Figure 8, each new potential configuration is checked by means of a holistic schedulability analyzer, following the guidelines described in Section 7.2.2, and a reliability analyzer. If the configuration meets all the requirements, it is output as a valid configuration. Otherwise, we can test the next potential configuration. The main advantage of this approach is that, if we have these two analyzers, it is very easy to implement. Moreover, if the non-functional requirements are checked by separated components, we have the flexibility to change those components at any time. Additionally, note that we could reduce the search execution time in two manners. On the one hand, we can execute the two analyzers in parallel and if one of them determines that the requirements are not met, the current iteration can be skipped. On the other hand, note from the figure that there is a flow of information from the different components to the beginning of the search process. The idea here is, upon the rejection of a configuration, gives some feedback to the first component to guide the generation of configurations in a smart manner. The main disadvantage of this approach is that if the component generating the configurations does not have any feedback, or the feedback is not useful, the configurations are generated in a random order and, depending on the space of configurations, it can take a huge amount of time.
2. The second search approach consists in including the real-time and reliability restrictions inside the search process. This makes it possible to discard invalid configurations earlier. Moreover, during the generation of a configuration, these restrictions are taken into account to allocate the tasks in the CNs, that is, the search process is driven by these restrictions. Consequently, the problem is converted into an optimization problem. The main advantage is that, for big configuration spaces, a valid configuration should be found in less time than with the previous approach. Moreover, we could consider that one valid configuration is better that another valid configuration, according to some criteria. For example, we could consider favoring configurations that are easier to reconfigure, configurations that have less energy consumption, or configurations that provide better fault tolerance. In this regard, it should be easy to integrate additional criteria during the search. The main disadvantage is that, in general, the implementation is more complex as we have to include the restrictions for the non-functional requirements manually. Moreover, they should be properly integrated into the search process. Furthermore, depending on the selected search algorithm, it could be even harder.

Now that we have presented the problem and discussed the two search techniques that we have considered, we can explain the experimental work we have carried out in this regard and the results we have obtained. Specifically, we have formulated a simplified version of the problem for which we have implemented and compared various search techniques. The problem we have considered consists of allocating a given number of tasks in a given number of nodes. Moreover, we modeled the real-time restrictions by assigning a cost to each task and a capacity to each node. A valid configuration is one that contains all the tasks and the aggregated cost of the tasks in the same node never surpasses its capacity. In our first work, we solved this problem using classical techniques like backtracking, heuristic-based techniques like branch and bound with a greedy algorithm, metaheuristic-based techniques like Tabu search [29], and solvers like Satisfiability Modulo Theories (SMT) solvers [30]. More details can be found in [31]. In a second work, we explored the use of machine learning. Specifically, we designed, built, and tested a model based on deep reinforcement learning that was able to produce valid configurations even faster than the typical heuristics used in problems similar to this one [32].

We are currently investigating on a proposal for a search solution that fulfills the different aspects stated at the beginning. Note, however, that depending on the size of the system, the search process could require a non-negligible amount of time. That is why we are also considering the use of proactive approaches. Specifically, instead of waiting for a change to occur and then starting the search process, we propose to foresee the changes that could affect the system and search for a valid configuration for each case in advance. Next, we list the approaches we could follow to trigger the search process:

- **Search when needed at runtime**: This is the classical reactive approach in which the search process is triggered by a change in the operational context.
- **Pre-search one level at runtime**: This approach takes the current configuration and determines all the possible changes in the operational context, or at least the most relevant ones that could occur. For each of them, the KE searches for a valid configuration and saves it for later use.
- **Pre-search all at runtime**: This approach is similar to the previous one, but instead of exploring just a single change in the operational context, we foresee multiple consecutive changes. The result of this is a tree in which the root is the current configuration and then, at the level *i*, we find valid configurations for the occurrence of *i* changes. Note that the depth of the tree could be established to any desired value.
- **Pre-search all offline**: In this approach, we assume an initial state of the system, and then we determine all the possible sequences of changes that could occur during the mission, and, for each one, we search a valid configuration. The result is a graph where the vertices are the valid configurations and the edges are the changes. Note that this is done offline and stored so it can be used during the operation of the system.

### 7.3. Configuration Change Process

When the KE determines that a reconfiguration is required, as shown in Figure 7, the new configuration is delivered to the *Configuration Change Manager* (CCM), which orchestrates the low-level changes. To carry out the changes related to the communications, the CCM relies on the *Main Communication Manager*. This module sends communication reconfiguration commands to the *Secondary Communication Manager* in the CNs to create, remove, or modify the attributes of the communications. Similarly, the CCM relies on the *Main Task Manager* to carry out the changes related to the tasks. This module sends task reconfiguration commands to the *Secondary Task Manager* in the CNs to start and stop tasks.

As we discussed in [33], the Configuration Change process starts by releasing all computational and communication resources that are no longer required according to the new configuration. It is noteworthy that this is a critical procedure since stopping tasks abruptly can leave the system in a unsafe state. Two aspects to consider in this regard are the false errors that could be detected during the transition and the state of the actuators.

On the one hand, stopping tasks without any specific order can provoke scenarios that can be interpreted as errors by the Monitoring Manager. For instance, if the communication resources are removed before the associated tasks are stopped, it will happen that a task will try to use these resources provoking, thus, an error, although this task is no longer needed. Consequently, tasks and their communication resources must be stopped taking into account their interdependencies and, in some cases, specific monitoring features must be disabled for the affected applications.

On the other hand, stopping tasks without any knowledge about the semantics of the applications can cause the state of the associated actuators to be wrong. For instance, when a semi-automatic vehicle switches from automatic to manual mode, it can be necessary to leave some of the actuators in a specific state so that the manual mode starts properly. The state in which the actuators associated with an application have to be left to perform a safe termination, we call it *termination condition*. In some applications, the termination condition consists in leaving the associated actuators in a predefined state, while in others consists in finishing the application cycle so that the state of the associated actuators is the last one calculated.

When all the computational and communication resources that are no longer required have been released, the CCM instructs the reservation of the new resources. To deal with the dependency issues, this is done in the opposite order of liberation. First, the new communications are created and then, the associated tasks are started. Note that neither the tasks nor the communications start to operate right after being created. More precisely, when all the computational and communication resources for a given application have been reserved, the NM triggers the execution of the associated tasks and the transmission of their messages in the appropriate order. This is possible since, as explained in Section 3, the NM maintains and enforces the scheduling for each application.

## 8. Feasibility

In this section, we demonstrate the feasibility of the design of the DFT4FTT infrastructure by presenting its implementation and showing its operation in various scenarios, each one involving a different change in the operational context.

As concerns the implementation of DFT4FTT, recall from Section 4 that it is composed of multiple interconnected hardware components, each one with its own requirements in terms of computer resources. The CNs and the SAs can be built using simple computing devices such as microcontrollers or more powerful embedded processors if the tasks to be executed require it. This is because the basic logic that implements the DFT4FTT features at the CNs and SAs requires a small amount of computational and memory resources. In contrast, the custom switch, together with its embedded NM, has more stringent computer resource requirements. This is because it is responsible for interconnecting and managing the operation of the CNs and the SAs. The device implementing this switch must be provided with multiple Ethernet interfaces and it must be able to carry out its operation with very low delay. As explained later in this section, we propose to build the switch using a regular multi-core computer equipped with enough Ethernet interfaces. Its logic, conversely, can be implemented in software. This provides a high level of flexibility to modify its behavior while having enough computational and memory resources to operate effectively. In fact, modern computers can be used to execute resource-intensive programs, such as programs for solving optimization problems. In this regard, using this type of device for implementing the switch is still a convenient decision even if its logic becomes more sophisticated. Note, however, that specific parts of the switch could be implemented using other devices, like a Field-Programmable Gate Array (FPGA), to reduce its response time when the system needs to operate under very tight time constraints.

Apart from the aforementioned implementation aspects, the amount of resources required to build the system directly depends on: the amount of DFT4FTT features finally implemented; the techniques used to implement them; the complexity of the application to be executed and the number of CNs and SAs the application requires.

To demonstrate the feasibility of the design of DFT4FTT we constructed a prototype composed of 1 NM and 6 CNs. Note that we tried to keep the physical architecture as simple as possible to focus on the fault tolerance capabilities of DFT4FTT. In this sense, this prototype did not cover de replication of the NM. Moreover, SAs are not directly connected to the NM. Instead, we assume that the specific sensors and actuators required in each scenario are already connected to the corresponding CNs.

This prototype includes all of the mechanisms that make it possible for the NM to: monitor the CNs to determine the state of the system; change its configuration, that is, the allocation of tasks; and, finally, enforce the corresponding scheduling of tasks and messages. Note that, as already explained in Section 7.2, as the definitive specification of the decision process is left for future work, in this experimental evaluation the set of changes that must be applied in each of the scenarios is statically defined.

The logic of the NM, as well as the CNs, was implemented in software using the C language, which is extensively used in embedded devices due to the high level of determinism it exhibits. In the case of the NM, the resulting software runs on a computer equipped with an Intel Core i7-4770 @ 3.40 GHz processor, 8 GB of RAM, and two Intel I350-T4 quad-port Ethernet server Adapters. In contrast, for the CNs, we use specific hardware typically used in embedded devices. Specifically, each CN is implemented by means of a Jetway JBC373F38-525-B barebone, which contains an Intel Atom D525 @ 1.80 GHz processor, 2 GB of RAM, and four standard Ethernet network adapters.

In the conducted experimentation, we reproduced the typical operational context changes under which an ADES operates. This includes:

- The initialization of the system
- Changes in the operational requirements due to a change in the mission phase
- Changes in the operational conditions due to permanent hardware faults
- Changes in the operational conditions due to changes in the environment

Regarding the software executed by the CNs, although DFT4FTT supports many different shapes of applications, to make the experimentation easy to construct, execute and explain in this prototype all the applications are shaped like a regular control application, which we believe is representative of the type of applications that are typically executed in an ADES. Recall from Figure 2a that a typical control application is composed of three tasks connected in the form of a chain. The execution cycle starts with task *S* which retrieves some sensor value that is sent to task *C* performing some processing on it to obtain an actuation value that is sent to task *A* which enforces it. Additionally, note that, as explained previously in Section 6.3, tasks may need to consult and modify data related to the state of the application and as tasks can be placed in any CN, said state must be exchanged among the tasks. That is why, every time a task sends a message to another one, the state of the application is piggybacked into said message. Moreover, the last task (the actuation task) sends the state of the application, and optionally the actuation value, to the first task (the sampling task) to close the cycle. Finally, the execution scheme of the application is comprised of 6 phases, each one involving the execution of a task (*S*, *C*, and *A*) or the transmission of their messages (data and state).

**Figure 9:**
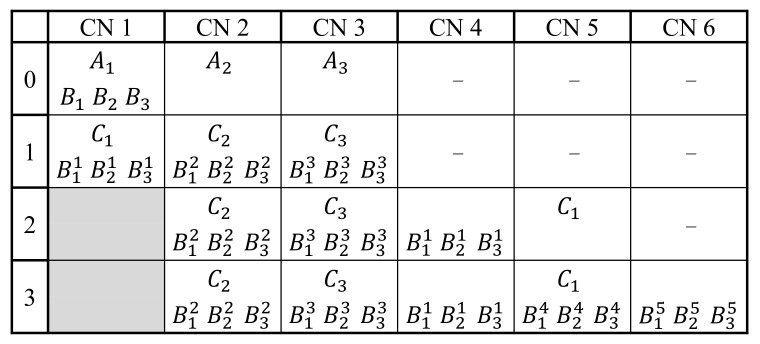
Experimentation scenarios.

Next, we describe each of the 4 scenarios involved in the experimentation we carried out. This includes the specific operational context simulated, as well as the response of the NM and the CNs. Figure 9 summarizes the configurations applied in each scenario. Each row corresponds to one specific scenario, while each column corresponds to one specific CN. Consequently, each cell of the table contains the list of tasks being executed in a given CN in a given scenario. Regarding the notation, the capital letter indicates the application, the subscript indicates the number of the task in said application and the superscript indicates the number of replicas. For instance, $B_{2}^{3}$ identifies the task 2 (control task) of application *B*, replica 3. Note that the number of replicas is omitted when no replication is used. The cells corresponding to the CNs that do not perform any task are depicted as “–”. Finally, grey cells denote faulty CNs.

### 8.1. Scenario 0: Initialization of the System

As soon as the hardware and software components have been powered on, the NM instructs the CNs to start running the necessary applications to cover the basic operations of the system. Specifically, as can be seen in Figure 9, in this prototype these applications are *A* and *B*. While the three tasks belonging to the application *A* are distributed in CNs 1, 2, and 3, the three tasks of application *B* are all executed in CN1.

This scenario demonstrates the ability of DFT4FTT to start a set of predefined applications when it starts automatically.

### 8.2. Scenario 1: Change in the Mission Phase

As explained in Section 3.2 the Mission Manager can modify the list of system requirements when a relevant event occurs. For this experimentation, we provide the Mission Manager with a mission specification indicating that the system must change to mission phase 1 when the value obtained by task $A_{1}$ through its sensor exceeds a certain threshold.

For this, the Mission Manager inspects the messages generated by CN1 and when the condition is met, the associated changes are written into the system requirements. This, in turn, triggers the execution of the KE, which instructs the CCM to apply the new configuration. Specifically, as shown in Figure 9, application *A* is deallocated in favor of application *C*, which is distributed in CNs 1, 2, and 3 identically as in the case of application *A*. Moreover, the reliability requirements of application *B* are increased and, as a result, the number of its task replicas is increased. More precisely, two new replicas for each task of application *B* are started in CNs 2 and 3.

This scenario demonstrates the ability of the NM to monitor the operation of the tasks to identify relevant events, stop and start applications, as well as increase the number of task replicas of an application from 1 to 3.

It is important to highlight several aspects regarding this scenario. First, all the changes for applications *A*, *B*, and *C* are triggered at the same time, at the end of the application cycle. This is possible since all of them have the same period and, thus, the end of the cycle coincides. However, nothing prevents the CCM from orchestrating individual changes in different phases of the cycle. Second, the mechanisms used to activate the replicated tasks and their communication have been designed to make said replication transparent. That is, it is not necessary to introduce special code to build a task replica. Finally, passing from 1 to 3 application replicas is not straightforward. This is because the state of the new replicas must be the same as the original replica. Moreover, this cannot be done through the regular reintegration mechanisms described in Section 6.3, as the number of new replicas is higher than the number of original replicas and, thus, the voting would mask the correct state. To overcome this issue, the NM initializes the new replicas in a particular manner. After loading the associated tasks into the CNs, the NM triggers the execution of all the replicas in the last phase of the application cycle. By doing this, the NM can intercept the message containing the correct state, transmitted by the actuation task of the original replica, and forward the corresponding message copies to the new replicas. During the first phase of the cycle, the new replicas update their state with the correct state received previously.

### 8.3. Scenario 2: Change in the Architecture

After the system automatically enforces the configuration associated with mission phase 1, we simulate the crash of CN1 by manually shutting it down. The NM is able to detect this failure due to the heartbeat mechanism described in Section 7.1.2. When the Monitoring Manager notices that CN1 omits the transmission of several consecutive IAA messages, CN1 is diagnosed as faulty. This triggers the execution of KE, which instructs the CCM to reallocate the tasks that were being executed in CN1 to other CNs. As shown in Figure 9, we move $C_{1}$ to CN5 and all the tasks of application *B*, replica 1, to CN4.

In this scenario, we demonstrate the ability of DFT4FTT to diagnose CNs as faulty and to reallocate tasks that have been lost to restore them. Note that, in the case of application *C*, this means that, after some downtime, its service is restored. However, in the case of application *B*, its service is still provided thanks to the additional replicas operating correctly. What the reallocation does is restore the lost replica to, in turn, restore its ability to tolerate additional faults in the future. Recall that this restoration process is possible thanks to the DFT mechanisms and it would not be possible with solely static ones.

### 8.4. Scenario 3: Change in the Environment

This experimentation finishes by simulating a more hostile environment. As a result, to maintain the required level of reliability of application *B*, the number of its task replicas is increased. For this, we simulate a radiation sensor connected to task $C_{1}$ and, thus, we can extract the level of harshness of the environment from the messages this task produces. Specifically, the Monitoring Manager supervises these message and updates the reliability level of all hardware components in the system state accordingly. Similarly to the previous cases, a modification in the system state triggers the execution of the KE which, in turn, instructs the CCM to increase the number of replicas of *B* from 3 to 5. As shown in Figure 9, the new replicas are started in CN5 and CN6.

Note that in this case, the increase in replication is completely transparent, as there is already a majority of replicas operating correctly. That is, the NM does not need to carry any additional operation to initialize the new replicas; instead, the regular reintegration mechanisms based on majority voting suffice to make the state of all the replicas consistent.

In this scenario, we demonstrate the ability of DFT4FTT to monitor the traffic to detect changes in the environment, update the system state accordingly, and increase the number of task replicas of an application from 3 to 5. Recall that this reconfiguration of the redundancy is only possible thanks to the DFT mechanisms. If only static ones were used, the number of replicas would have been 5 from the beginning which is not efficient.

## 9. Limitations

DFT4FTT presents interesting features that make it suitable for implementing a wide range of ADES. Note, however, that during the design some concessions were made to achieve these features and, thus, it does not suit in some specific cases. The most important limitation of DFT4FTT is derived from its use of a star topology. Having the NM in the center of the network has several benefits: (1) the traffic can be monitored and controlled very easily, (2) the process of finding a new system configuration is easier since the topology is very simple, (3) changing the system configuration is straightforward and takes a short time as the CNs are directly connected to the NM, and (4) the design of the fault tolerance is much simpler, as it can be implemented in a centralized manner. Nevertheless, if the system to be built requires CNs and SAs to be highly distributed, for instance, in a smart city, the amount of necessary cabling would make this endeavor unattainable. Likewise, the maximum number of CNs and SAs a DFT4FTT-based system can handle is also constrained. This is because the NM needs to dedicate specific network hardware and computational resources to each component connected to it. Consequently, a large number of components may require an amount of hardware and resources impossible to place inside the switch.

A system requiring high distribution and/or a large number of nodes has to be designed using a more appropriate topology. In these cases, it is typical to adopt a mesh topology composed of multiple switches interconnecting the nodes. Moreover, in networks like those, the Software-Defined Networking (SDN) paradigm is typically used to ease the management of the switches. For this, SDN follows a centralized approach in which one or more SDN controllers (the control plane) dynamically reconfigure the forwarding tables of the multiple switches (the data plane). Despite SDN was not initially designed for industrial application, due to its lack of real-time and dependability support [34,35], in the last years, there have been many works, like these ones [36,37,38], trying to overcome these limitations. Consequently, although SDN does not address the management of the tasks, it is a potential solution that could be adapted to implement the communication subsystem of DFT4FTT. Despite that, FTTRS natively ensures a real-time and reliable behavior, and as explained above, provides several benefits that ease the design and implementation.

Another aspect that limits the self-reconfiguration capabilities of DFT4FTT is the number of hardware resources. In this regard, note that these capabilities are built on top of non-physical components like tasks and messages. These elements can be created, removed, and modified dynamically. In contrast, physical components like CNs and network links are also necessary, but they cannot be managed dynamically. When the system is started, a specific amount of physical resources are available, and they gradually degrade due to permanent faults. Since these resources cannot be regenerated, the self-reconfiguration of DFT4FTT is constrained by the ones that are available at each instant.

It should be mentioned that, although we did not address the *maintainability* [20] of the system, that is, the “ability to undergo modifications and repairs”, the flexibility with which we designed DFT4FTT could be used to implement additional mechanisms to support the replacement of CNs and SAs, with little effort.

## 10. Conclusions and Future Work

In this paper, we describe the work we carried out to design and implement the fundamental aspects of a self-reconfigurable infrastructure to build distributed embedded systems with real-time, reliability, and adaptivity requirements. At the network level, this infrastructure relies on FTTRS, a network that makes it possible for the nodes to exchange messages while guaranteeing the three previously mentioned requirements. Specifically, FTTRS provides a means to exchange real-time traffic while allowing to change online the attributes of the communications. Moreover, it provides high reliability through space and time redundancy. At the node level, this infrastructure implements the functionalities in the form of applications. Each application has specific real-time and reliability requirements and is composed of tasks executed in a sequential and/or parallel manner. We have designed a set of mechanisms that make it possible to dynamically allocate tasks in the nodes performing the computation. A given allocation of tasks and messages, together with their operational attributes, is called configuration, and a change in the configuration can be triggered by a change in the system requirements or in the system state.

To ensure that real-time requirements are fulfilled, every time a new configuration is needed, the system searches for a configuration that makes all the tasks and messages meet their deadlines. As concerns the reliability requirements, critical tasks are replicated using the N-Modular Redundancy with spares technique. Consequently, when searching for the new configuration, we also ensure that critical tasks have enough replicas.

One of the most important aspects to address in future work is the single point of failure that the Node Manager represents. To solve this issue, we plan to duplicate the Node Manager and introduce mechanisms to make both replicas replica-determine so that they can operate in parallel and seamlessly tolerate the failure of one of them.

Another aspect we are working on is the characterization of the self-reconfiguration time. This is a very important system attribute from a real-time perspective as it determines how fast the system can react to changes. As explained, the self-reconfiguration is done in three steps: detect the need for a new configuration, determine a new valid configuration and apply the said configuration. While the first and the third steps are deterministic and, thus, we can find an upper bound, we do not yet have any mechanism to determine how much time it takes to find a valid configuration using the searching approaches discussed.

From a dependability perspective, it is also very appealing the idea of extending the decision process and the fault tolerance mechanisms of DFT4FTT to support multiple fault tolerance strategies that could be applied dynamically. This would make it possible to give support to other dependability attributes such as availability or safety.

Finally, note that the name of the Knowledge Entity is not arbitrary. The purpose of this software component is to collect information about the system and, by means of a set of rules (the knowledge) decide on the system configuration. In this regard, we also considered the idea of complementing it with a so-called Wisdom Entity that, instead of rules, could operate by means of machine learning. In contrast to the Knowledge Entity, which follows a reactive approach, the Wisdom Entity could follow a proactive approach in which the artificial intelligence could foresee the need for changes in the configuration and instruct them in advance to maximize the efficiency and effectiveness of the system.

## Figures and Tables

**Table 1:** Taxonomy of the DFT4FTT fault-tolerance mechanisms.

Type	Technique	Mechanism	Origin
Static Fault Tolerance	Error compensation	Spatial replication of the channel	R
		Temporal replication of critical messages	R
		Task Replication with majority voting	R
	Error recovery	Forward error recovery	R
	Reintegration of Lost Redundancy	Periodic triggering of task execution and message transmission	A
		Reintegration of task replica internal state through voting	A
	Reintegration of very Lost Redundancy	Software reset and Reintegration	R
		Hardware reset and Reintegration	R
Dynamic Fault Tolerance	Restoration of Lost Redundancy	Reallocation of task	N
	Reconfiguration of the Redundancy	Adjust number of message replicas	N
		Adjust number of task replicas	N
	Reconfiguration of the Fault Tolerance	Select the proper fault-tolerance strategy	N

## Data Availability

Not applicable.

## Acronyms

The following acronyms are used in this manuscript:

ADES	Adaptive distributed embedded system
CCM	Configuration Change Manager
CEC	Communication Error Counter
CN	Computational Node
COTS	Commercial Off-The-Shelf
DEC	Discrepancy Error Counter
DES	Distributed embedded system
DFT	Dynamic Fault Tolerance
DFT4FTT	Dynamic Fault Tolerance for the Flexible Time-Triggered Ethernet
EC	Elementary Cycle
FTT	Flexible Time-Triggered communication paradigm
FTTRS	Flexible Time-Triggered Replicated Star
KE	Knowledge Entity
MM	Monitoring Manager
NIC	Network Interface Controller
NM	Node Manager
NMR	N-Modular Redundancy
PG	Port Guardian
SAs	Sensors and actuators
SDN	Software-Defined Networking
TM	Trigger Message
